# A new mission for an old anti-cancer drug: harnessing hepatocyte-specific metabolic pathways against liver tumors

**DOI:** 10.1038/s41392-023-01513-5

**Published:** 2023-06-14

**Authors:** Weiting Liao, Diego F. Calvisi, Xin Chen

**Affiliations:** 1 Department of Medical Oncology, Cancer Center, West China Hospital, Sichuan University, 610041 Chengdu, China; 2 Cancer Biology Program, University of Hawaii Cancer Center, Honolulu, 96813 Hawaii USA; 3 Institute of Pathology, University of Regensburg, Regensburg, 93053 Germany

**Keywords:** Gastrointestinal cancer, Cancer therapy

In a recent study published in *Nature Cancer*, Shi et al. reported the identification of the small molecule YC-1 as the selective drug against primary liver tumor cells due to the specific expression of sulfotransferase family 1A member 1 (SULT1A1) in hepatocyte-lineage cells.^1^ This study offered new insights into repurposing an old anti-cancer drug via harnessing hepatocyte-specific metabolic enzymes to treat primary liver tumors.

Primary liver cancer, mainly consisting of hepatocellular carcinoma (HCC) and intrahepatic cholangiocarcinoma (iCCA), is a leading cause of cancer-related deaths worldwide. Due to the lack of specific symptoms, most HCC and iCCA cases are diagnosed at the advanced stage, limiting treatment options. Currently, immune checkpoint inhibitor (ICI)-based combination therapies have become the first-time treatment options for advanced-stage HCC and iCCA. However, two-thirds of patients do not respond to ICI therapies.^2^ Therefore, there is an urgent need to identify novel targeted treatments. In this study, the authors performed high-throughput pharmacologic screens to identify novel small molecules that could target the deadly disease.

The initial screening was toward *Isocitrate dehydrogenase 1 (IDH1)*-mutant iCCA. *IDH1* is one of the most frequently mutated genes in iCCA. The mutant form of IDH1 acts as an oncogene to promote iCCA development by blocking cell differentiation and suppressing anti-tumor immunity.^3^ The high-throughput screening was conducted on 1912 oncology-focused compounds against two *IDH1* mutant iCCA and two *IDH1* wild-type cell lines. The screen identified 36 small molecules effective against *IDH1* mutant iCCA cells. Among them, the most effective compounds against *IDH1* mutant iCCA were SRC family kinase inhibitors and YC-1. SRC family kinase inhibitors are known to suppress the growth of *IDH1* mutant cancer cells. Therefore, the authors chose to focus on YC-1 as the candidate compound.

YC-1 was discovered about 20 years ago as a hypoxia-inducible factor 1-α (HIF1α) inhibitor.^4^ However, other HIF1α inhibitors failed to suppress *IDH1* mutant iCCA cell growth. Consistently, CRISPR-mediated deletion of HIF1α and HIF2α showed no tumor inhibitory activities against *IDH1* mutant iCCA cells. Although the underlying reasons for this phenotype remain undefined, the data indicates that YC-1 suppresses *IDH1* mutant iCCA cell growth via a distinct mechanism. Next, the authors tested YC-1 against a panel of 26 cholangiocarcinoma cell lines. The experiment confirmed that all *IDH1* mutant iCCA cells were sensitive to YC-1. However, several *IDH1* wild-type iCCA cells were also susceptible to the drug, suggesting that YC-1 operates independently of *IDH1* mutation status. To solve this mystery, the authors expanded their studies on YC-1 against 1022 cancer cell lines derived from over 25 tumor types. Among them, ~10% of the cell line collection was susceptible to the anti-neoplastic activity of YC-1. Remarkably, the sensitive cell lines mainly consisted of primary liver cancer cells, including HCC and iCCA cell lines.

To identify the molecular mechanisms underlying YC-1’s selective activities, the authors developed RBE cells with acquired resistance to YC-1 by increasing YC-1 concentration in the culture, followed by mass spectrometry analysis. Subsequently, the authors revealed the loss of the SULT1A1 protein in YC-1-resistant RBE cells. Further investigation confirmed that high SULT1A1 expression is associated with sensitivity to YC-1. Notably, the deletion of SULT1A1 via CRISPR-Cas9 mediated gene editing in YC-1 sensitive cell lines triggered resistance to YC-1. In contrast, overexpression of SULT1A1 in YC-1-resistant cells conferred sensitiveness. These data indicate that SULT1A1 determines sensitivity to YC-1 in cancer cells.

**Fig. 1:**
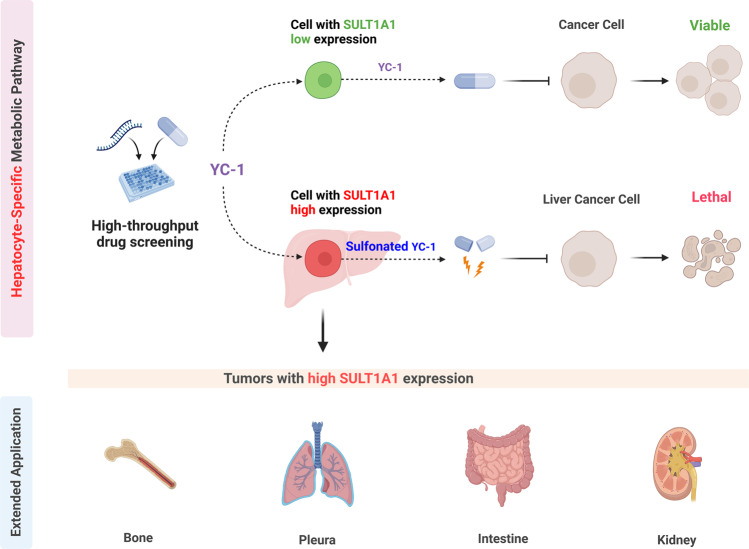
The anti-neoplastic potency of YC-1 depends on SULT1A1 status in liver tumors. The high-throughput pharmacologic screens conducted by Shi et al. identified the synthetic lethal interactions of YC-1 with specific liver cancer subtypes overexpressing the cytosolic phenol sulfotransferase SULT1A1 (cells with high SULT1A1 expression). Specifically, SULT1A1, physiologically expressed by cells of the hepatocellular lineage, metabolically activates the YC-1 prodrug by sulfonation. Once sulfonated, YC-1 becomes vigorously active against liver cancer cells. This event does not occur in liver tumors with low SULT1A1 expression. Similarly, YC-1 or analogous compounds could represent a new potential avenue for treatment opportunities in other SULT1A1-activated tumors. Created with BioRender.com

SULT1A1 is a phenol sulfotransferase, highly expressed in hepatocytes, regulating xenobiotic metabolism.^5^ Intriguingly, iCCA cells sensitive to YC-1 display the unique protein expression program enriched in hepatocyte markers. Next, using immunohistochemistry, the authors revealed strong or intermediate SULT1A1 in most human iCCA and HCC specimens. Further chemical and biochemical studies showed that YC-1 is, in fact, a prodrug, and SULT1A1 sulfonates it. Once sulfonated, YC-1 binds to and alkylates multiple proteins in the cell. Using chemoproteomic assays, the authors identified 250 proteins that bind to sulfonated YC-1. Pathway enrichment analysis revealed that RNA-binding proteins, which regulate RNA metabolism, splicing, and translation, were significantly enriched. These genes are known to be required for cell survival. These studies demonstrate that sulfonated YC-1 targets RNA-binding proteins, leading to tumor cell death. Finally, subcutaneous and orthotopic xenograft models and SULT1A1-high or low expression CCLP1 iCCA cells were investigated. As expected, YC-1 treatment was effective against SULT1A1-high CCLP1 cells in vivo. In summary, the present data reveal that YC-1 or similar compounds might be therapeutically helpful against SULT1A1-expressing neoplasms, especially primary liver tumors. (Fig. 1)

The study is highly significant and has great translational potential. Importantly, it provides a novel treatment strategy via harnessing hepatocyte-specific metabolic enzymes to treat primary liver tumors. In addition to YC-1, the study identified additional small molecules showing similar SULT1A1-dependent anti-tumor activities. These compounds should also be further investigated for their therapeutic potential in primary liver cancers. It is also fascinating that a subset of iCCA expresses both cholangiocyte and hepatocyte markers, in this case, SULT1A1. The cellular origin of iCCA is still under debate.^6^ Lineage tracing and genomic profiling studies have suggested that cholangiocytes, hepatocytes, and/or progenitor-like cells can give rise to iCCAs. The current study provides additional evidence that iCCA is a heterogeneous disease possibly originating from various cell types. It is tempting to speculate that those iCCAs developed from hepatocytes and/or progenitor-like cells will maintain SULT1A1 expression and, therefore, might be the target of YC-1 or similar compounds. Besides the primary liver cancers, YC-1 was also effective against tumors from bone, pleura, intestine, kidney, etc. Thus, the therapeutic efficacy of YC-1 in these tumor types should be tested both in vitro and in vivo (Fig. 1).

Besides its merits, the manuscript also displays some limitations. While YC-1 in mice seems to be well-tolerated, the long-term effects of these molecules remain to be determined. In physiologic conditions, the liver, intestine, lung, and adrenal glands express SULT1A1. Therefore, the outcome of YC-1 treatment on these organs should be further elucidated. For instance, how YC-1 affects hepatocyte homeostasis needs to be thoroughly analyzed, as YC-1 will be sulfonated in normal hepatocytes, and sulfonated YC-1 will bind to proteins in the normal hepatocytes. This effect may disrupt liver homeostasis, leading to hepatic toxicity. The in vivo studies in the current manuscript are somehow limited and do not address this critical issue. Expanding the studies using genetic mouse HCC and iCCA models would be necessary. In addition, investigations with human HCC and iCCA PDX models and organoids will be helpful to elucidate better the therapeutic efficacy of YC-1 in vivo. Furthermore, therapeutic approaches to reactivate SULT1A1 should be investigated to render SULT1A1-negative tumors targetable by YC-1. Finally, drug screening should be conducted to discover additional compounds synergizing with YC-1 against cancer growth and progression.

In summary, the study by Shi et al. identified YC-1 as a SULT1A1-dependent anti-tumor compound, providing a novel and promising biomarker-based targeted therapy against primary liver cancers.
